# Zinner syndrome: Clinical insights from Western Norway

**DOI:** 10.1177/03915603251335609

**Published:** 2025-05-13

**Authors:** Emanuel Bjurulf, Lars A R Reisæter, Hemamaalini Rajkumar, Adeel Asghar Chaudhury, Alfred Honoré, Florin Hopland-Nechita, Christian Arvei Moen, Julie Nøss Haugland, Ravi Rawal, Ingunn Roth, Anh Khoi Vo, Christian Beisland, Patrick Juliebø-Jones

**Affiliations:** 1 Department of Urology, Haukeland University Hospital, Bergen, Norway; 2 Department of Radiology, Haukeland University Hospital, Bergen, Norway; 3 Department of Fertility Medicine, Haukeland University Hospital, Bergen, Norway; 4 Department of Clinical Medicine, University of Bergen, Bergen, Norway; 5 Department of Urology, Førde Central Hospital, Førde, Norway

**Keywords:** Zinner syndrome, ejaculatory duct obstruction, renal agenesis, MRI, male infertility

## Abstract

**Introduction:**

Zinner syndrome (ZS) is characterised by unilateral renal agenesis, ipsilateral seminal vesicle cyst and obstruction of the ejaculatory duct. Although rare, urologists may encounter it at some point in their clinical practice. The literature is largely limited to case reports, and the condition is poorly understood. Our objective was to report on cases of ZS that have been managed at two centres in order to gain further clinical insights on this condition.

**Methods:**

A retrospective review was conducted on ZS cases presenting at two centres in Western Norway between January 2021 and June 2024. Data were collected on demographic details, symptomatology, imaging findings, management and fertility outcomes.

**Results:**

Six cases were identified that met the full triad for ZS, with ages ranging from 18 to 70 years. Five patients were symptomatic at presentation, reporting issues such as anejaculation and testicular pain during sexual activity. Two cases presented as emergencies, one with acute urinary retention and the other with severe pelvic pain. Half of the patients were successfully managed with a conservative approach. Two-thirds had children, either through natural conception or assisted reproductive methods, while the remaining patients underwent sperm cryopreservation.

**Conclusion:**

ZS presents with a wide range of symptoms and at varying ages. Not all symptomatic cases require surgical intervention, and management should be individualised. In select cases, a conservative approach can feasible.

## Key messages

- Zinner syndrome can display varied symptomology and age at presentation.
- Not all symptomatic cases will necessarily require surgical intervention.
- Further studies are needed to investigate the impact of Zinner syndrome on fertility.

## Introduction

Zinner syndrome (ZS) is characterised by the clinical constellation of unilateral renal agenesis, ipsilateral seminal vesicle cyst and obstruction of the ejaculatory duct.^1^ Known to be rare, the true incidence and prevalence of this urogenital malformation remains unknown. However, with the increasing use of cross-sectional imaging, a rise in the number of diagnosed ZS cases could be expected. The anomaly originates during the first trimester of pregnancy due to the abnormal development of the mesonephric (Wolffian) duct.^2^ Typical symptoms that are associated with this congenital condition include urinary and ejaculatory complaints. The parallel embryonic abnormality in females, known as Herlyn-Werner-Wunderlich Syndrome, results in ipsilateral renal agenesis and uterus didelphys with an obstructed hemivagina. This condition can lead to complications such as endometriosis and spontaneous abortion.^3^

While ZS was first described over 100 years ago, the literature on this condition remains limited, primarily consisting of case reports.^4^ Our objective was to report on cases of ZS managed at two centres in order to gain further clinical insights on this condition.

## Methods

A retrospective review was performed of ZS cases presenting to two centres in Western Norway between January 2021 and June 2024. To be included in the study, patients were required to have the complete triad of unilateral renal agenesis, ipsilateral seminal vesicle cyst and obstruction of the ejaculatory duct. Three cases were excluded as they met only two of the three diagnostic criteria. Data collected included demographic details, symptomology, imaging findings and management. In addition, information on fertility status was also gathered. All patients provided informed consent for inclusion in the study.

## Results

Over a 3-year period, six cases of ZS were identified, with the age at diagnosis ranging from 18 to 70 years (Table 1). Except for one case, all diagnoses were confirmed via magnetic resonance imaging (MRI). Five of the six patients were symptomatic at presentation, all of whom were under 50 years old. The two patients reporting sexual dysfunction were both in their second decade of life. One patient reported testicular pain during sexual activity, while the other presented with persistent anejaculation that had lasted for several years. Two of the symptomatic cases had presented as an emergency; one due to acute urinary retention and the other due to acute pelvic pain. The only asymptomatic case involved an elderly male who was found to have an elevated prostate specific antigen (PSA) during routine blood testing in the community and subsequently underwent MRI, which revealed the diagnosis of ZS. Interestingly, a review of historical records revealed that the anatomical features of ZS had been visible on cross-sectional imaging performed at another hospital over a decade earlier. However, these radiographic findings had not led to a diagnosis at the time. In this case, a biopsy confirmed prostate cancer (PCa), and the patient subsequently underwent radical prostatectomy. While ZS was determined to be unrelated to his PCa, the distorted anatomy did result in a technically more challenging operation. None of the cases had any significant past medical history of note. In all cases, following urological assessment, a diagnosis of ZS was established within a maximum of 4 months. Not all cases required surgical intervention, with a conservative approach being successfully implemented in half of the patients. Regarding fertility status, half of the cases had already fathered children via natural conception prior to their presentation. The remaining cases were referred to a fertility centre for further evaluation and management. Among the latter, two patients underwent sperm cryopreservation due to their young age, while the third successfully conceived a child through assisted reproductive methods. In that particular case, the patient and their partner had been unable to conceive naturally for several years before the patient’s acute presentation with pain.

**Table 1.** Summary of cases and findings.

Case no.	Age at presentation	Symptomatic at presentation	Emergency?	Symptoms	PMH	Investigations	Management	Fertility Status
1	18 years	Yes	No (Cancer referral pathway)	Testicular pain during sexual activity	Nil	US (Figure 1) CT	Orchidectomy due to suspected malignancy. Negative. Histology showed cystic dilatation of rete testis and epididymis	Referred to fertility centre. Sperm cryopreservation performed.
2	18 years	Yes	No (Normal referral pathway)	Anejecaulaton Normal erection and libido Mild scrotal discomfort	Nil	Cystoscopy MRI CT	Conservative	Referred to fertility centre. Sperm cryopreservation performed.
3	45 years	Yes	No (Normal referral pathway)	Scrotal pain. Treatment for suspected epididmitis. US picked up single kidney	ITP	US MRI	Conservative	Offspring prior to presentation via natural conception.
4	46 years	Yes	Yes	Acute urinary retention	GORD	US MRI	Conservative	Offspring prior to presentation via natural conception.
5	34 years	Yes	Yes	Pelvic and scrotal pain	Nil	CT MRI	Surgery - Robotic excision of seminal vesicle cyst	Referred to fertility centre. Successful conception with assisted reproductive methods
6	70 years	No	No (Cancer referral pathway)	Asymptomatic. Raised PSA	Nil	MRI CT	Robotic radical prostatectomy for biopsy confirmed prostate cancer	Offspring prior to presentation via natural conception.

PMH: past medical history; US: ultrasound; CT: computed tomography; MRI: magnetic resonance imaging; PSA: prostate specific antigen; ITP: idiopathic thrombocytopaenia; GORD: gastro-oesophageal reflux disease.

**Figure 1.**
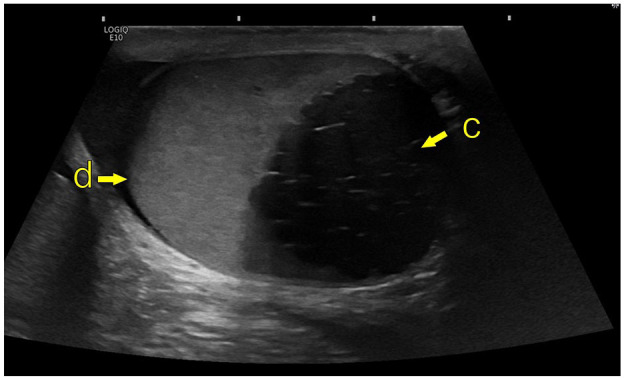
Ultrasound male genital organs; GE Logique10 (GE HealthCare); linear transducer ML6-15. (d) Left testicle, (c) Cystic enlargement of the left seminal vesicle.

## Discussion

The true epidemiology of ZS is complex and difficult to establish. Firstly, it is not covered in the International Classification of Diseases (ICD) system, making searches in local and national health registries not possible to perform. One potential approach is to manually identify cases within male patients registered under the ICD-10 code Q60.0 for *Unilateral Renal Agenesis*, though this method remains limited in scope.

Many individuals with the characteristic pathognomic triad may remain asymptomatic, and as a result, the underlying condition may go undiagnosed. It is possible therefore that ZS may not be as rare as generally considered. With the increasing use of cross-sectional imaging and the rising detection of incidental findings across various conditions, a higher rate of new ZS diagnoses could be expected. At the same time, however, this does not seem to have occurred despite the increase in MRI imaging, which has become an established part of the diagnostic pathway for suspected PCa.^5^ Given that the syndrome is rare, potential new diagnoses may be missed if a clinician or radiologist is unaware of the pathology. A 10-year review of published cases of ZS in China, a country with a population exceeding 1.4 billion, revealed only 16 documented cases.^1^ Therefore, identifying six cases within a population of less than one million in Western Norway over a 3-year period is arguably quite significant. Bearrick and Husmann proposed that young men diagnosed with a congenital solitary kidney should be monitored over time to assess for the potential development of ZS.^6^

ZS usually presents from the second decade of life onwards, coinciding with the onset of sexual and reproductive activity. Azoospermia is believed to result from ejaculatory duct obstruction. Approximately half of the patients with ZS reported in the literature are documented to be infertile.^2^ However, given that ZS is characterised by unilateral abnormalities only, a definitive answer is lacking as to why patients should be infertile if the contralateral anatomy is normal. Suggested theories include chronic obstruction leading to the release of toxic and reactive oxygen species and/or anti-sperm antibody production.^7^ However, most studies lack semen analysis results and instead rely solely on patient-reported fertility status, limiting the accuracy of infertility assessments. Indeed, the interplay between ZS and fertility remains one of the most poorly understood aspects of the condition. Beyond the aforementioned challenges, several other key questions remain unanswered, which highlights the need for further research. For example, it remains unclear whether symptomatic ZS is more strongly associated with infertility compared to asymptomatic cases. While this was not observed in our series, other research groups have suggested this.^7^ In those who have conceived naturally, the absence of semen analysis data makes it unclear what proportion may still have impaired sperm quality compared to the general population. Additionally, it remains unknown whether surgical intervention can effectively reverse fertility impairments in affected individuals.

**Figure 2.**
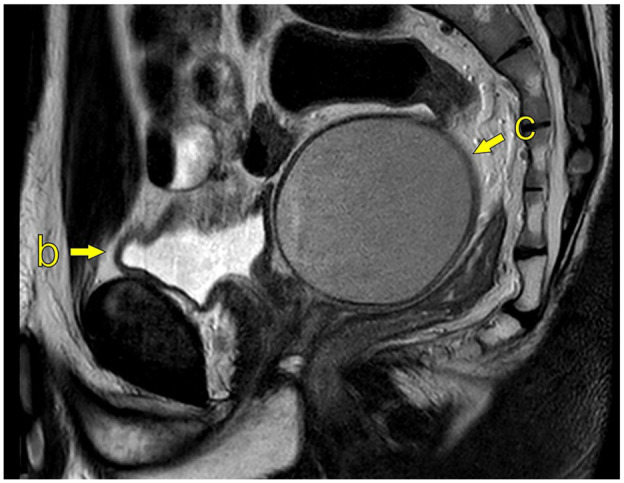
MRI of the pelvis; 1.5T Philips Ingenia (Philips Medical System), T2W sagittal image (TR 4000/TE 80). (b) Bladder, (c) Cystic enlargement of left seminal vesicle, measuring 7.2 × 6.7 × 9.1 cm (ca. 219 mL).

**Figure 3.**
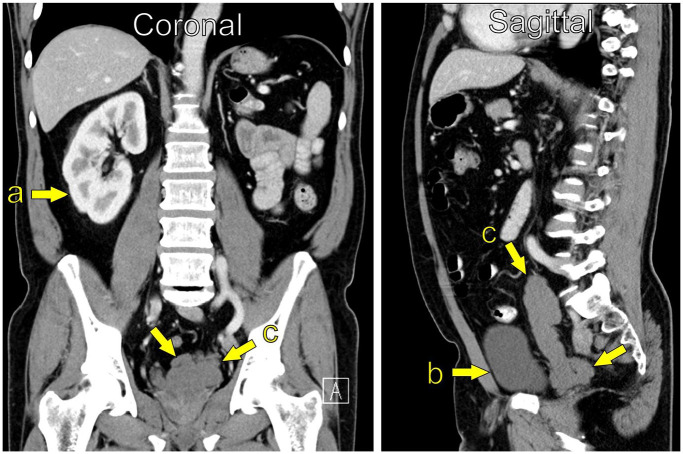
CT Abdomen/pelvis (Coronal and Sagittal reconstruction); Siemens Definition AS (Siemens Healthineers); unknown i.v. contrast-agent, unknown injection volume and injection rate. (a) Single kidney right side, (b) Bladder, (c) Cystic enlargement of the seminal vesicle, measuring 3.4 × 3.0 × 12.2 cm (ca. 62 mL).

**Figure 4.**
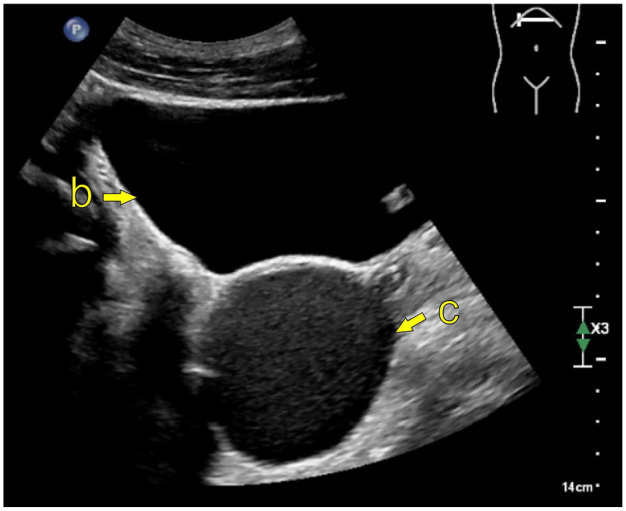
Transabdominal ultrasound; EPIQ Elite (Philips Medical System); curved transducer C5-1. (b) Bladder, (c) Cystic enlargement of the left seminal vesicle.

MRI is considered the imaging modality of choice (Figure 2), but findings associated with ZS can often be detectable on computed tomography (CT; Figure 3). Ultrasound may also indicate features of ZS, but it is operator-dependent, and more clinical acumen is needed (Figure 4).

The management plan for ZS is dictated on a case-by-case basis. This is a common conclusion in reported studies and aligns with our findings. Notably, for the clinician faced with a case of ZS, who may seek to consult established protocols, the management of ZS receives no mention or recommendation in international guidelines. Clinical guidance is therefore largely limited to expert opinion based on findings from smaller studies. While our study is indeed limited by its retrospective status, the findings were gathered across two centres. A Norwegian ZS registry has been proposed to build on these findings and serve as a platform to address knowledge gaps. It could serve as an advantageous means to facilitate infertility and genetic assessments and thus shed light on an area of ZS that is poorly understood.

## Conclusion

ZS can display varied symptomology and age at presentation. Not all symptomatic cases will necessarily require surgical intervention. Management should therefore be determined on a case-by-case basis. A conservative approach can be feasible in select cases. Prospective studies are needed to address knowledge gaps, particularly concerning the impact of ZS on fertility.
